# PtRTP1, an Effector of *Puccinia triticina*, Contributes to Virulence in Wheat

**DOI:** 10.3390/plants15192973

**Published:** 2026-09-29

**Authors:** Jiaying Chang, Zijia Zhang, Ju Zhang, Liuning Xuan, Hongfei Yan, Na Zhang, Wenxiang Yang

**Affiliations:** 1 Henan Key Laboratory of Crop Molecular Breeding and Bioreactor, Henan Province Engineering Research Center of Plant Gene and Molecular Breeding, Key Laboratory of Plant Genetics and Molecular Breeding, Zhoukou Normal University, Zhoukou 466001, China; changjiaying@163.com (J.C.); zjtt603@126.com (J.Z.);; 2 Technological Innovation Center for Biological Control of Plant Diseases and Insect Pests of Hebei Province, College of Plant Protection, Hebei Agricultural University, Baoding 071000, China; 20252130449@pgs.hebau.edu.cn (Z.Z.); hfy2008@hebau.edu.cn (H.Y.)

**Keywords:** *Puccinia triticina*, effector, PtRTP1, virulence function, wheat leaf rust

## Abstract

Effector proteins are key virulence factors of obligate parasitic fungi during infection. Wheat leaf rust by *Puccinia triticina* is a widespread destructive disease drastically limiting wheat production, while the biological role of its effector is still elusive. PtRTP1 is a homolog of Uf-RTP1p, one of the earliest effector proteins verified to localize at the host–pathogen interface. Clarifying PtRTP1 function helps uncover rust pathogenic mechanisms; hence, we cloned PtRTP1 from *P. triticina*. The gene has a 1453 bp DNA sequence and a 777 bp CDS, encoding a protein with a 23 aa signal peptide, nine cysteines and two N-glycosylation sites. PtRTP1 was differentially expressed during infection, peaking at 24 hpi. Its expression pattern was consistent across races, with higher levels in the highly virulent strain THTT. Analysis of the nucleotide-sequence polymorphism of PtRTP1 from nine physiological races of *P. triticina* revealed two polymorphic sites. Heterologous expression assays demonstrated that the PtRTP1 protein could suppress BAX-induced programmed cell death and functioned in the nucleus. Host-induced gene silencing revealed that silencing PtRTP1 weakened fungal pathogenicity, reducing pustules and infection areas in wheat lines TcLr15 and TcLr36. This indicates secreted PtRTP1 acts as a virulence factor.

## 1. Introduction

Wheat, the world’s third major staple crop, suffers severely from various diseases that pose a great threat to global food security. Wheat leaf rust threatens 94.4% of global wheat production and is one of the most destructive diseases causing substantial wheat yield losses worldwide [1,2]. Wheat leaf rust, caused by the airborne phytopathogenic fungus *Puccinia triticina* Erikss., is a major destructive foliar disease of wheat. Severe outbreaks can result in yield losses of more than 15% [1,3]. *P. triticina* is a highly specialized obligate biotrophic fungus that depends on living host tissues for growth and reproduction [4,5]. During infection, the pathogen has evolved mechanisms to evade or suppress plant defense responses and regulate host metabolic pathways to sustain its survival and propagation [6]. This fungus obtains nutrients from host cells via haustoria. Apart from absorbing carbohydrates, haustoria secrete a large number of effector proteins, which enable the rust fungus to inhibit multiple host immune responses [7,8,9]. Therefore, clarifying the functions of effector proteins is essential to unravel their pathogenic mechanisms and provide a theoretical basis for the development of novel fungicide targets.

Research on effector proteins of *P. triticina* is still in its infancy. To date, more than 2000 candidate effectors have been identified by means of whole-genome sequencing, comparative genomics and transcriptome analysis [10,11,12,13,14,15,16]. Transcriptomic databases of three *P. triticina* single-spore isolates (KHTT, JHKT and THSN) at different infection stages were further constructed, from which 635 candidate effector proteins were precisely screened out [12]. Preliminary progress has been made in the functional characterization of these effectors. Five avirulence genes have been reported so far. The effector Pt13024 confers avirulence to wheat line TcLr30 [17]. The avirulence gene *AvrLr21* modulates *Lr21*-mediated immune responses [18]. Pt1641 exhibits avirulent activity in the near-isogenic wheat line TcLr1 [19]. Natural mutations of *AvrLr15* enable the pathogen to evade recognition by the wheat resistance gene *Lr15* [20]. The effector Pt_20636 is a bifunctional protein: it acts as a virulence effector to suppress host resistance against leaf rust by disrupting the TaLhcb4.1-mediated reactive oxygen species (ROS) pathway; meanwhile, it likely functions as the avirulence gene *AvrLr16* and regulates wheat immune signaling via direct or indirect interaction with immune receptors encoded by *Lr16* [21]. Several effectors with clarified action mechanisms have also been documented. The effector Pt_1234 targets a subdomain C of wheat transcription factor *TaNAC069* and thereby impairs *TaNAC069*-mediated defense responses against leaf rust [22]. Pt3372 suppresses the expression of defense-related genes *TaPR1* and *TaPR2* by targeting TaERP3, which attenuates wheat immune responses [23]. The chloroplast-localized thaumatin-like effector Pt9029 interacts with wheat Rubisco activase (TaRCA) via its TLP domain. This interaction inhibits TaRCA activity, reduces Rubisco activity, and ultimately weakens wheat resistance to leaf rust [24]. Nevertheless, the functions and molecular mechanisms of numerous candidate effectors remain uncharacterized. Therefore, further exploration and functional validation of candidate effectors have become a major research focus in this field.

The trafficking and subcellular localization of effectors are essential for their biological functions during the interaction between rust fungi and host plants. As the pivotal structure for nutrient acquisition and effector secretion in rust fungi, haustoria are the key research objects to decipher the pathogenic mechanisms of rust pathogens [8,25,26]. Among the reported rust fungal effectors, the RTP family has attracted increasing research attention due to its unique trafficking properties and biological roles during infection. Members of this family are among the first fungal effectors proven to be translocated into host cells, and they are specifically distributed in *Pucciniales*. Their distinct structural and functional features provide valuable clues for elucidating the action modes of *P. triticina* effectors, with relevant studies originating from the pioneering work conducted by Hahn and Mendgen in 1997 [8,27,28].

Hahn and Mendgen identified Uf-RTP1p, a rust transfer protein 1, from *Uromyces fabae* via screening of in planta-induced genes (PIGs). At the early stage of haustorium development, Uf-RTP1p mainly accumulates in the extrahaustorial matrix. As infection proceeds, this protein is gradually detected in the cytoplasm and nucleus of infected host cells [27]. By contrast, Us-RTP1p from *U. striatus* is also localized to the extrahaustorial matrix and host cytoplasm, but is barely detectable in the nucleus of infected plant cells [28]. Both Uf-RTP1p and Us-RTP1p predominantly exist in a glycosylated form in infected leaves [28]. Previous studies have confirmed that RTP1p can form amyloid-like filaments both in vitro and in vivo [29]. Although RTP family members are widely present in rust fungi, they are exclusively found in *Pucciniales*. Structural analysis reveals that RTP1p homologs consist of an unstructured N-terminal domain and a structured C-terminal domain, and possess proteinase inhibitor activity [30]. As a homolog of Uf-RTP1, the effector Pst18363 from *P. striiformis* f. sp. tritici targets TaNUDX23, a protein involved in ROS production. This interaction inhibits ROS accumulation and facilitates the infection of *P. striiformis* [31]. *P. triticina* belongs to the order *Pucciniales*; however, no homologs of RTP1 have been reported in this pathogen, and its roles during infection remain uncharacterized.

In the present study, PtRTP1, a homolog of Uf-RTP1, was cloned from *P. triticina*. To characterize the basic properties of PtRTP1 in *P. triticina*, we performed qRT-PCR to analyze the expression patterns of PtRTP1 in two physiological races (THTT and FGD) of *P. triticina*. Heterologous expression assays in *Nicotiana benthamiana* were conducted to examine the subcellular localization of PtRTP1 as well as its suppressive effect on BAX-induced programmed cell death (PCD). To further elucidate the function of PtRTP1 during *P. triticina* infection, we employed host-induced gene silencing (HIGS) to silence PtRTP1 in two wheat near-isogenic lines (TcLr15 and TcLr36), and investigated the roles of PtRTP1 in the infection process of different virulent races (THTT and FGD) of *P. triticina*. This study is expected to improve our knowledge of *P. triticina* pathogenicity and help establish whether PtRTP1 acts as a candidate virulence determinant.

## 2. Results

### 2.1. Cloning and Expression Profiling of PtRTP1 from Puccinia triticina

**Figure 1:**
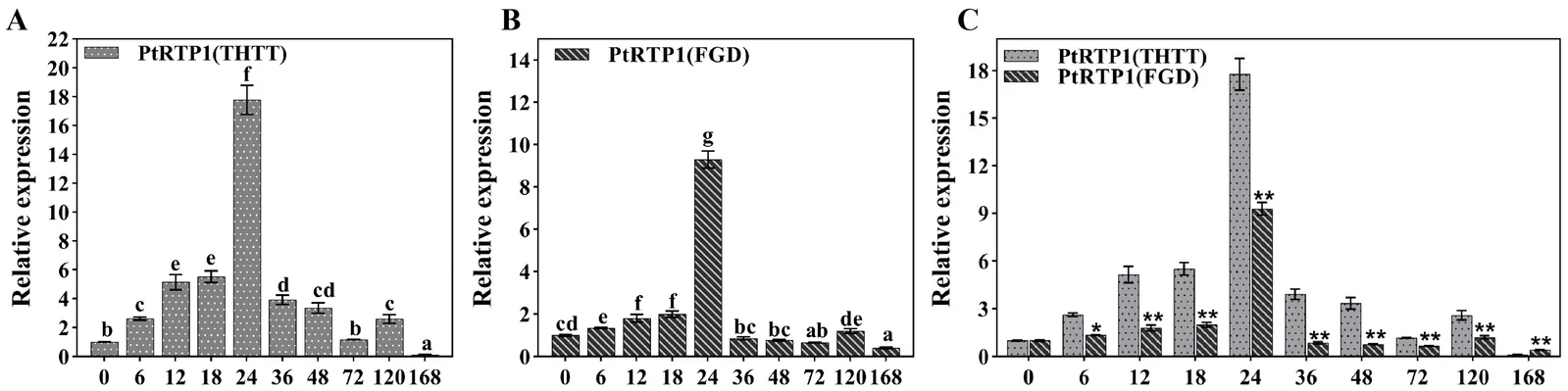
Expression patterns of *PtRTP1* among different physiological race of *Puccinia triticina*. (**A**) Expression pattern of PtRTP1 in the THTT race of *P. triticina*. The expression patterns of *PtRTP1* were assessed using RNA extracted from urediniospores, and leaves of wheat cultivar Thatcher were inoculated with *P. triticina* at various time points, including 6, 12, 18, 24, 36, 48, 72, 120, and 168 h post inoculation (hpi). The relative expression levels were determined using the comparative 2^−ΔΔCt^ method. Standard error and mean fold changes were calculated based on data obtained from three independent biological replicates. Different letters indicate significant differences in *PtRTP1* transcript levels across different time points post inoculation of *P. triticina* (*p* < 0.05, analysis of variance). (**B**) Expression pattern of PtRTP1 in the FGD race of *P. triticina*. Different letters indicate significant differences in *PtRTP1* transcript levels across different time points post inoculation of *P. triticina* (*p* < 0.05, analysis of variance). (**C**) Asterisks denote comparisons of *PtRTP1* expression levels between the highly virulent strain THTT and the low-virulence strain FGD (* *p* < 0.05, ** *p* < 0.01, unpaired two-tailed Student’s *t*-test).

The target fragment was amplified from cDNA derived from the interaction between *P. triticina* and wheat cultivar Thatcher using primer pair PtRTP1-F/PtRTP1-R, yielding a band of approximately 750 bp. Sequencing confirmed that the open reading frame (ORF) of PtRTP1 was 777 bp in length, and the full-length sequence of PtRTP1 of *P. triticina* was successfully obtained. qRT-PCR was performed to characterize the transcriptional patterns of PtRTP1 at distinct infection stages in two *P. triticina* isolates with contrasting virulence, THTT (highly virulent) and FGD (weakly virulent), during infection of Thatcher. The results revealed that PtRTP1 transcription was induced upon pathogen inoculation and peaked at 24 hpi, followed by a gradual decline, with negligible expression detected after 168 hpi (Figure 1A,B). The two isolates exhibited nearly identical expression patterns of PtRTP1, yet transcript abundance was consistently higher in THTT than in FGD across all tested time points (Figure 1C).

### 2.2. Sequence Polymorphism of PtRTP1

**Figure 2:**
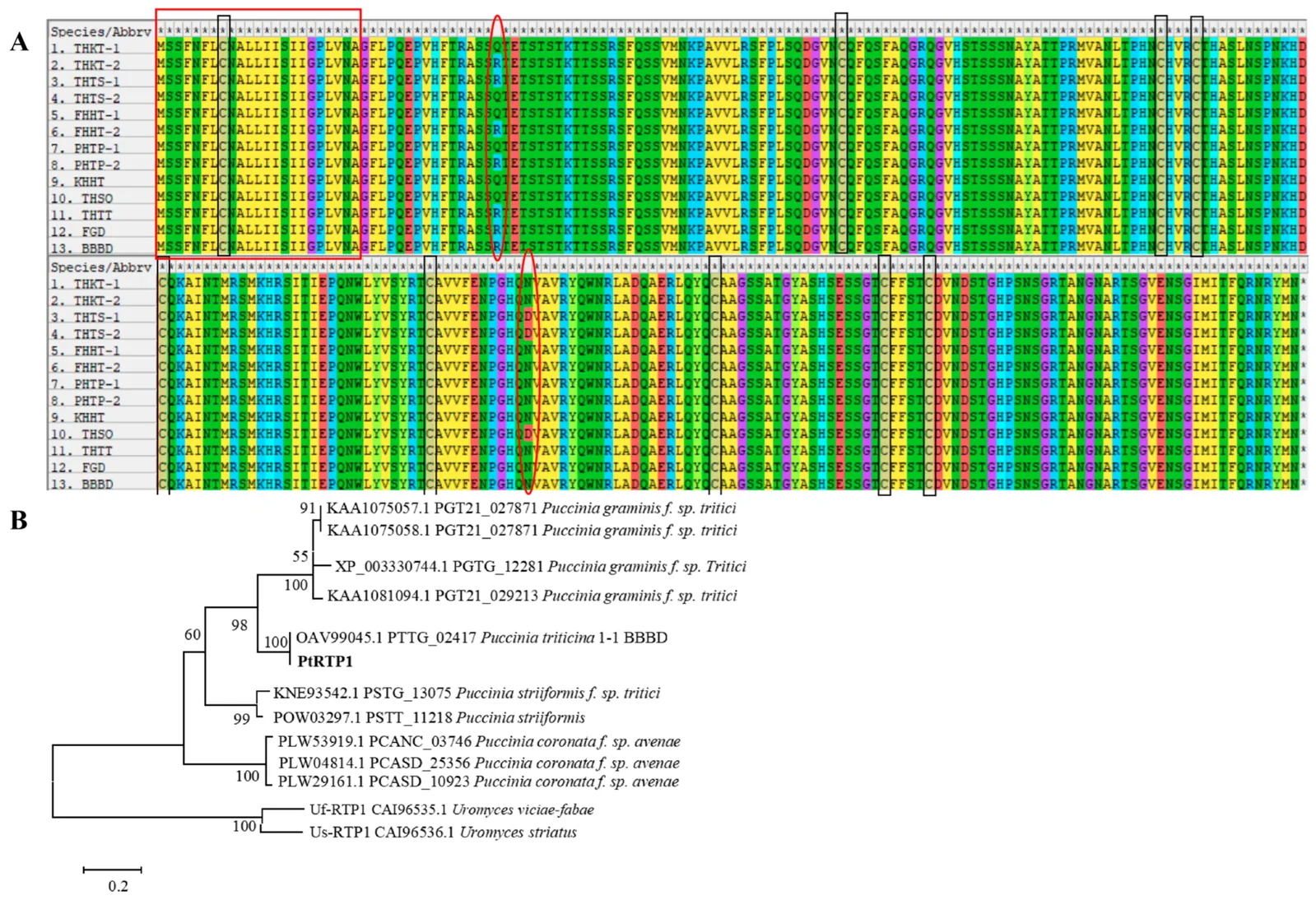
Analysis of PtRTP1 amino acid sequence polymorphism. (**A**) The red box highlights the signal peptide region. The black box delineates conserved cysteine residues. The red ovals demarcate polymorphic sites. Asterisks (*) indicate identical amino acids at the corresponding position, whereas positions without asterisks show amino acid differences. (**B**) Evolutionary analysis of PtRTP1 in *Puccinia triticina*.

The genomic sequence of *PtRTP1* in *P. triticina* spans 1453 bp, consisting of nine exons and eight introns. Signal P 6.0 prediction identified a 23 aa signal peptide (marked by the red box in Figure 2A). The theoretical molecular weight of the unprocessed precursor protein is 28.3 kDa, while the mature protein has a predicted mass of 25.9 kDa. Two putative N-glycosylation sites (N108 and N220) were predicted within the PtRTP1 protein. Nucleotide-sequence polymorphism of PtRTP1 was characterized across nine *P. triticina* physiological races, and two polymorphic sites were detected. All nine isolates contained a polymorphic site at nucleotide position 116 (G/A), which led to an amino acid substitution at residue 39 (arginine R/glutamine Q, red ovals in Figure 2A). A second variant site at nucleotide 514 (A/G) exclusively existed in races THTS and THSQ, causing an amino acid change at position 172 (asparagine N/aspartic acid D, red ovals in Figure 2A). Nine conserved cysteine residues (C8, C80, C125, C129, C142, C172, C204, C225, C230) were conserved among all 13 sequences (black boxes in Figure 2A).

Homologous sequences of PtRTP1 protein were retrieved from the NCBI database, and a total of nine homologs belonging to the genus *Puccinia* were obtained, including *P. coronata* f. sp. *avenae*, *P. graminis* f. sp. *tritici* and *P. striiformis* f. sp. *tritici*. Phylogenetic analysis revealed that PtRTP1 was distantly related to RTP1 homologs from *P. striiformis* f. sp. *tritici* and *P. coronata* f. sp. *avenae* (bootstrap support = 60), whereas it shared the closest phylogenetic relationship with RTP1 from *P. graminis* f. sp. *tritici* (bootstrap support = 100) (Figure 2B).

### 2.3. The Signal Peptide of PtRTP1 Possesses Secretory Activity

**Figure 3:**
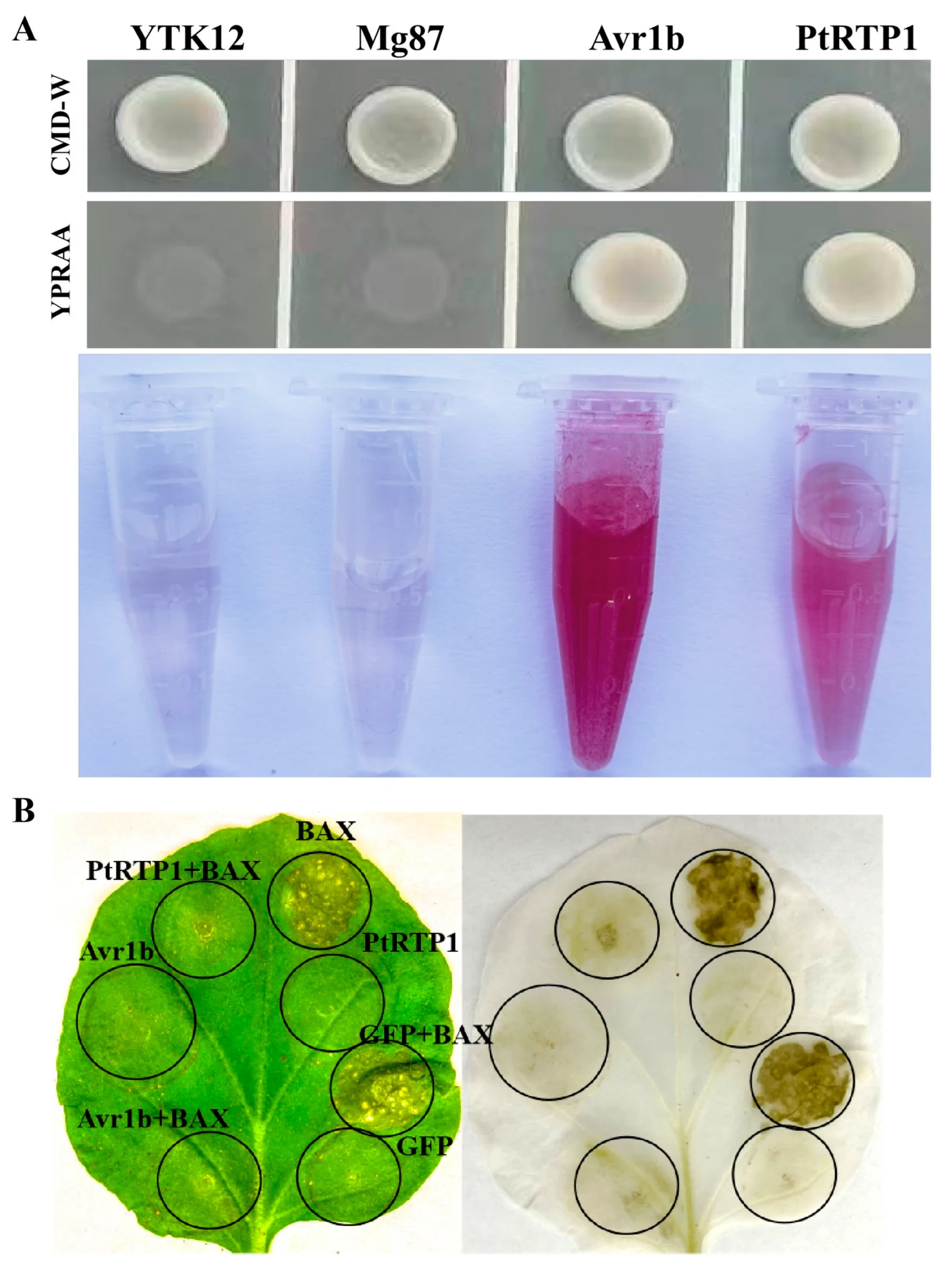
Secretory function of the PtRTP1 signal peptide and its inhibition of BAX-induced programmed cell death. (**A**) Analysis of the secretory function of the PtRTP1 signal peptide. Signal peptides endowed with secretory properties facilitate the extracellular release of invertase, catalyzing the reduction of 2,3,5-triphenyltetrazolium chloride to insoluble triphenylformazan (red). The visual manifestation of the red hue validates the secretory function of the signaling peptide. Avr1b served as the positive control, while Mg87 and YTK12 functioned as the negative controls in the experimental setup. (**B**) Analysis of the inhibitory effect of PtRTP1 on BAX-induced programmed cell death in *Nicotiana benthamiana*.

The TTC reduction assay based on 2,3,5-triphenyltetrazolium chloride was used to detect secreted invertase activity and verify the secretory function of the PtRTP1 signal peptide. YTK12 strains harboring Mg87 and the empty vector served as negative controls, whereas the YTK12 strain harboring Avr1bSP was used as the positive control. Strains YTK12 carrying Avr1bSP (positive control) and PtRTP1SP were capable of exporting invertase extracellularly to hydrolyze sucrose into monosaccharides. Monosaccharides subsequently reacted with TTC to generate insoluble red triphenylformazan. In contrast, the negative control Mg87 exhibited no secreted invertase activity. These results demonstrate that the signal peptide of PtRTP1 is functional for protein secretion (Figure 3A).

### 2.4. PtRTP1 Suppresses Plant Immune Responses

BAX can trigger hypersensitive cell death in *N. benthamiana*, and this phenotype was utilized to test whether PtRTP1 suppresses host immunity. Similar to the *oomycete* effector Avr1b (positive control), PtRTP1 markedly inhibited BAX-triggered PCD. By contrast, the empty vector pGR107:GFP (negative control) showed no inhibitory effect on BAX-induced PCD. Chlorophyll removal from leaf tissues with ethanol facilitated clearer visualization of cell death lesions. Collectively, these data indicate that PtRTP1 functions as an immune suppressor (Figure 3B).

### 2.5. Subcellular Localization of PtRTP1 in N. benthamiana

**Figure 4:**
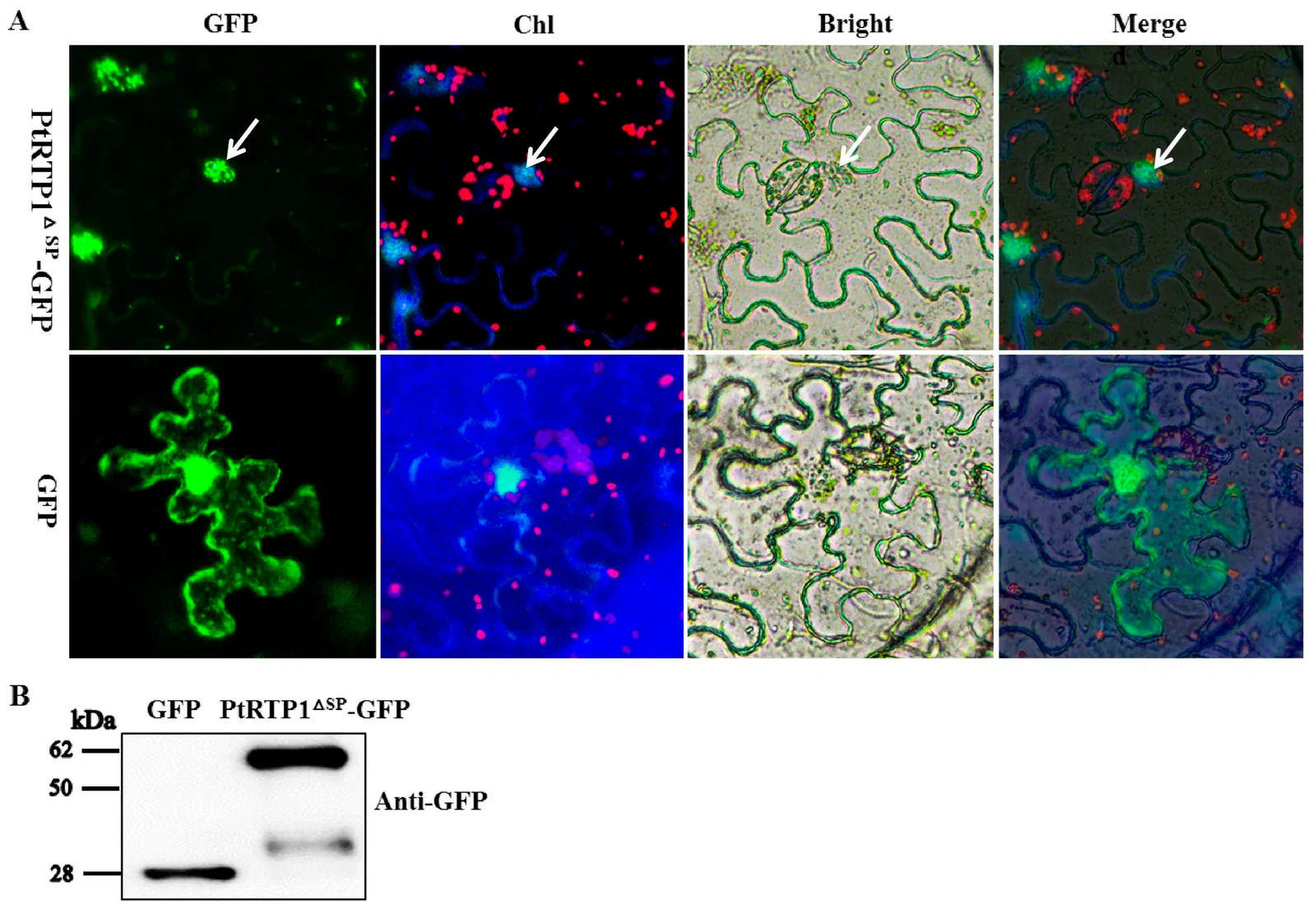
Subcellular localization of PtRTP1. (**A**) Heterologous fusion expression of PtRTP1, GFP: fluorescence field; Bright: bright; Merged: merged field; GFP-pGR107: positive control; PtRTP1-pGR107: experimental group; scale = 50 μm. The white arrows indicate the nucleus. (**B**) Western blot was used to detect the expression of PtRTP1.

To identify the intracellular action site of PtRTP1, empty pGR107 was used as the control. *Agrobacterium tumefaciens* carrying pGR107 or pGR107-PtRTP1^△sp^ was infiltrated into *N. benthamiana* leaves, and fluorescence signals were observed under a fluorescence microscope at 48 h post infiltration. Leaves expressing empty pGR107 displayed GFP signals throughout the whole cell, while strong green GFP fluorescence from pGR107-PtRTP1^△sp^ was concentrated in intracellular organelles, mainly the nucleus, confirming successful expression and nuclear localization of PtRTP1^Δsp^ (Figure 4A). Total protein was extracted from infiltrated *N. benthamiana* leaves for Western blot analysis using anti-GFP antibody. The fusion protein PtRTP1^Δsp^-GFP exhibited a molecular weight of approximately 50 kDa, consistent with the theoretical size (26 kDa for GFP plus 25 kDa for PtRTP1). This result verified the successful expression of PtRTP1 in plant cells (Figure 4B).

### 2.6. PtRTP1 Acts as a Virulence Effector

**Figure 5:**
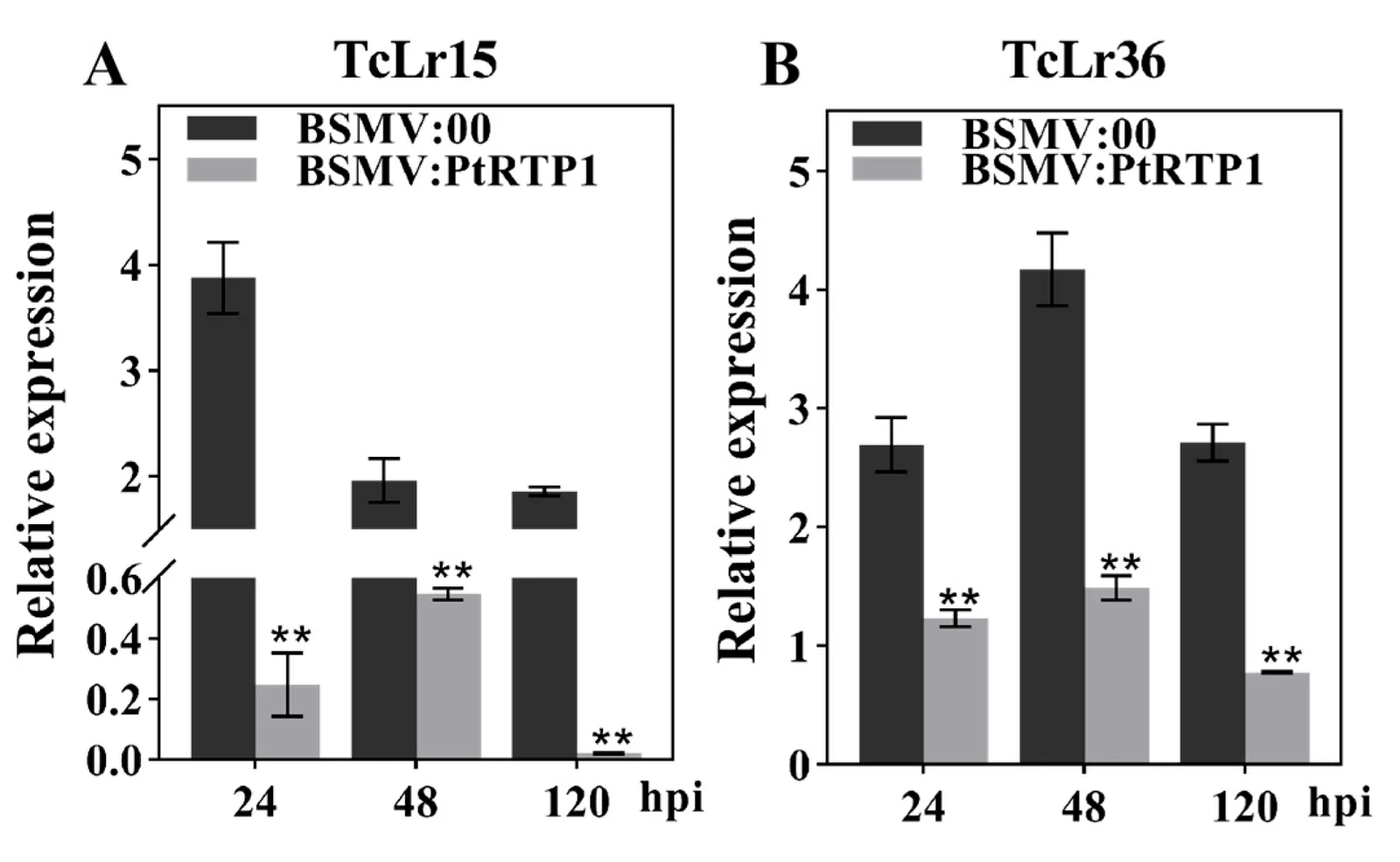
The silencing efficiency of *PtRTP1* was assessed using qRT-PCR. (**A**) Silencing efficiency of *PtRTP1* in TcLr15. (**B**) Silencing efficiency of *PtRTP1* in TcLr36. There were 3 biological replicates for each treatment; significant difference by unpaired two-tailed Student’s t-test. The asterisks indicate significant difference in samples with *PtRTP1* silenced by HIGS in comparison with the control (** *p* < 0.01).

**Figure 6:**
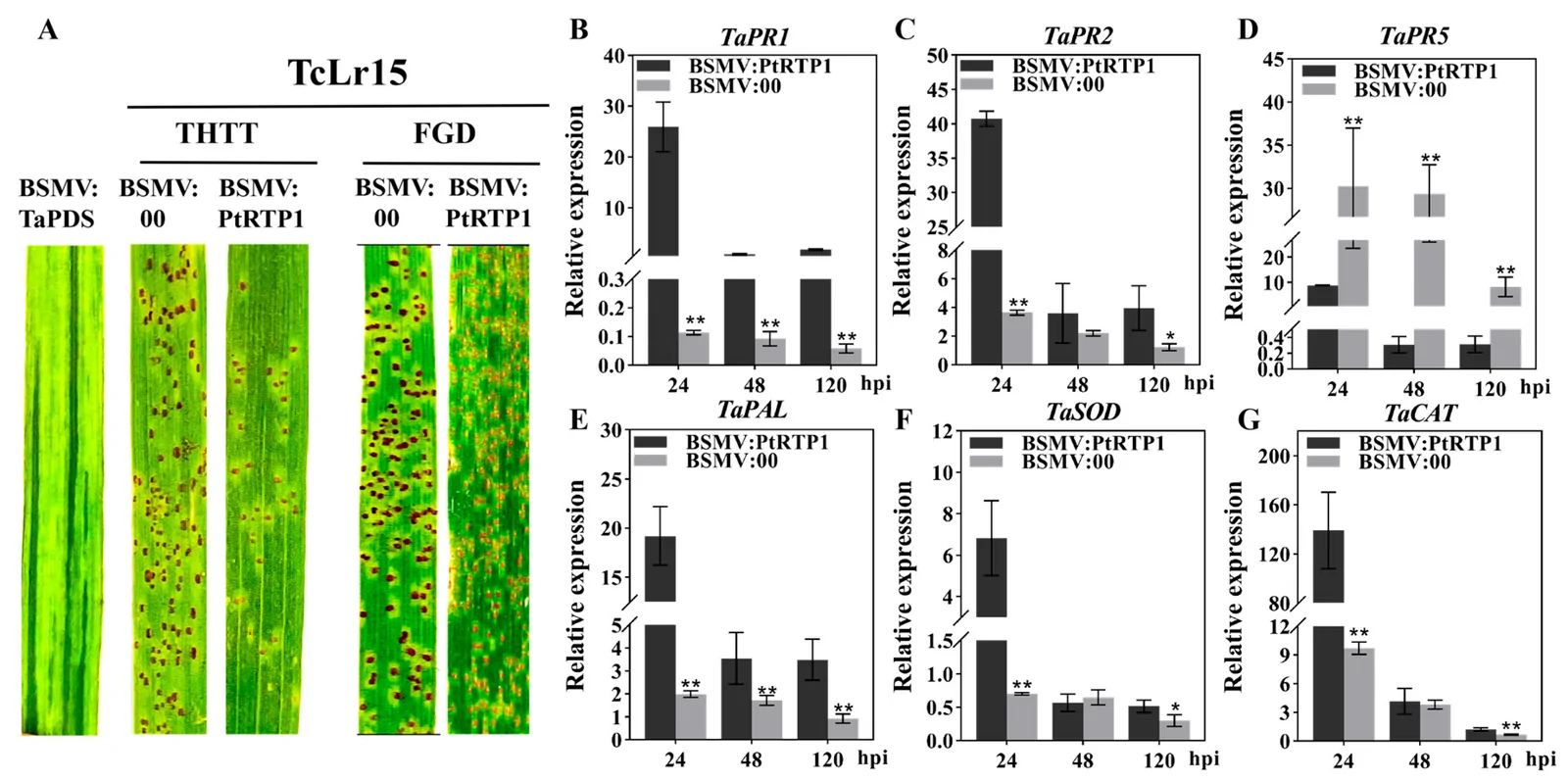
Silencing of *PtRTP1* attenuated the virulence of *Puccinia triticina* on TcLr15. (**A**) Pathogenic phenotypic analysis of *P. triticina* after silencing *PtRTP1*. (**B**–**G**) The relative expression levels were determined using the comparative 2^−ΔΔCt^ method. Standard error and mean fold changes were calculated based on data obtained from three independent biological replicates. Asterisks denote comparisons of *TaPR1*, *TaPR2*, *TaPR5*, *TaPAL*, *TaSOD*, and *TaCAT* expression levels between the BSMV:PtRTP1 and the BSMV:00 (* *p* < 0.05, ** *p* < 0.01, unpaired two-tailed Student’s *t*-test).

**Figure 7:**
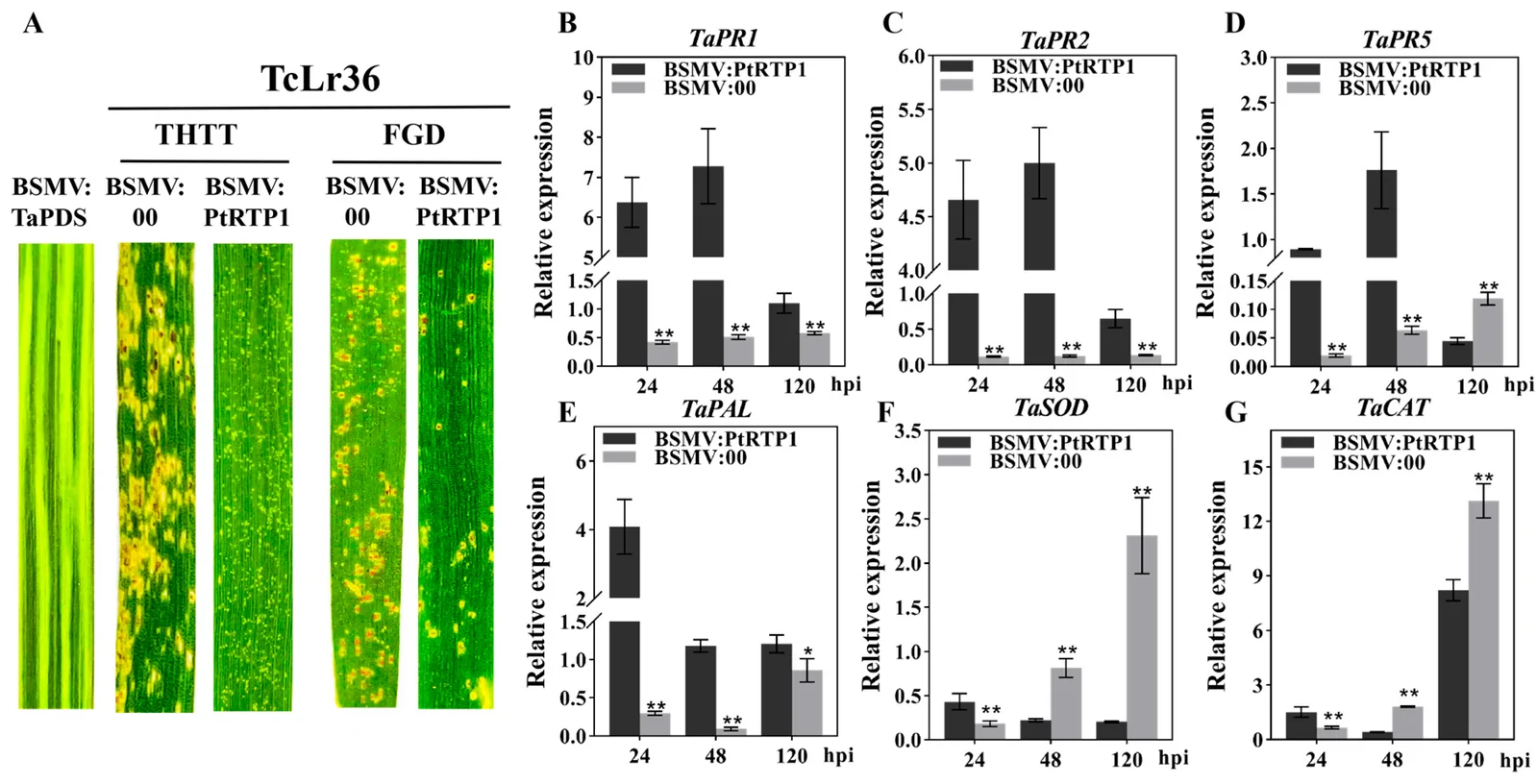
Silencing of *PtRTP1* attenuated the virulence of *Puccinia triticina* on TcLr36. (**A**) Pathogenic phenotypic analysis of *P. triticina* after silencing PtRTP1. (**B**–**G**) The relative expression levels were determined using the comparative 2^−ΔΔCt^ method. Standard error and mean fold changes were calculated based on data obtained from three independent biological replicates. Asterisks denote comparisons of *TaPR1*, *TaPR2*, *TaPR5*, *TaPAL*, *TaSOD*, and *TaCAT* expression levels between the BSMV:PtRTP1 and the BSMV:00 (* *p* < 0.05, ** *p* < 0.01, unpaired two-tailed Student’s *t*-test).

Barley stripe mosaic virus (BSMV)-mediated host-induced gene silencing (HIGS) was performed to explore the role of PtRTP1 during *P. triticina* infection. qRT-PCR results showed that transcript levels of *PtRTP1* were markedly suppressed at 24, 48 and 120 hpi in TcLr15 and TcLr36 wheat plants pre-inoculated with BSMV:PtRTP1 compared with the empty vector control BSMV:00, verifying the effective silencing of *PtRTP1* (Figure 5). Wheat seedlings inoculated with BSMV:TaPDS displayed obvious photobleaching at 14 dpi (Figure 6A). Mild mosaic yellowing was observed on plants treated with BSMV:PtRTP1 at the same time point, confirming successful viral infection. Fourteen days after inoculating *P. triticina* onto virus-treated wheat, virulence attenuation was observed for both THTT and FGD when *PtRTP1* was silenced. On TcLr15, silencing of *PtRTP1* drastically reduced uredinial density for THTT and shrank uredinial size for FGD (Figure 6A). But there was no significant change in its infection type; all are “2”. On TcLr36, THTT only produced tiny infection sites without visible uredinia, while FGD formed far fewer uredinia relative to control plants. The infectious type changed from “1” and “2” to “;” and “1” respectively (Figure 7A). These data demonstrate that PtRTP1 contributes to full virulence of both tested physiological races of *P. triticina*.

qRT-PCR was further used to quantify transcript abundance of defense-related genes in TcLr15 and TcLr36 with silenced PtRTP1 in race THTT. In TcLr15, silencing PtRTP1 significantly upregulated *TaPR1*, *TaPR2*, *TaPAL*, *TaSOD* and *TaCAT*, whereas *TaPR5* was downregulated (Figure 6B–G). Combined with disease phenotypes, we hypothesize that repressed PtRTP1 impairs the colonization and proliferation of THTT and thereby restricts lesion development. Since *TaPR5* promotes programmed cell death to block pathogen spread, its low expression may explain the residual small lesions observed on TcLr15. In TcLr36, the transcripts of *TaPR1*, *TaPR2*, *TaPAL* and *TaPR5* were significantly elevated, while *TaSOD* and *TaCAT* were suppressed (Figure 7B–G). Phenotypic observations suggest that silencing PtRTP1 inhibits pathogen reproduction and uredinia formation yet triggers cell death at infection sites. The decreased expression of *TaSOD* and *TaCAT* weakens cellular antioxidant capacity, which may trigger oxidative damage and subsequent localized cell necrosis at infection foci.

## 3. Discussion

RTP1 is the first identified secreted rust protein proven to translocate directly from haustoria into host cells during infection [28]. This protein forms fibrillar structures within the extrahaustorial matrix and host cytoplasm. It is hypothesized that these fibrils protect haustoria and mediate tight attachment between plant nuclei and haustoria to sustain intimate pathogen–host contact. Notably, it is unusual for a single protein to possess both structural and enzymatic functions [29]. In this study, the RTP1 homolog PtRTP1 was cloned from *P. triticina*. qRT-PCR analysis revealed consistent transcriptional patterns of PtRTP1 during wheat infection by two races with distinct virulence (THTT and FGD). The gene was transcriptionally induced post inoculation and peaked at 24 hpi, a time point coinciding with haustorium formation in *P. triticina*. Given that Uf-RTP1 generates fibrillar materials in the extrahaustorial matrix [29], we propose that PtRTP1 also participates in haustorium biogenesis and is critical for maintaining haustorial stability of this obligate biotrophic pathogen.

Since RTP1 was characterized in *U. fabae* and *U. striatus* [28], numerous RTP1 homologs have been identified across genera of all suborders within *Pucciniales*. RTP1 homologs are relatively conserved among rust fungi, while no homologous sequences have been detected in other fungal lineages. This indicates that the RTP1p family is rust-specific, which is presumed to be associated with their unique obligate biotrophic lifestyle [30]. Numerous physiological races of *P. triticina* have been identified, yet the conservation of PtRTP1 across these races remains unreported. In this study, sequence polymorphism analysis of PtRTP1 from nine *P. triticina* races uncovered two polymorphic amino acid sites, giving rise to four distinct sequence variants of this gene. However, the biological impact of these polymorphic variants remains uncharacterized. Using HIGS, we preliminarily confirmed that PtRTP1 acts as a virulence factor required for the infection process of both THTT and FGD, likely facilitating pathogen colonization and urediniospore production. The functional significance of these *PtRTP1* sequence polymorphisms is currently unknown; future studies will perform functional comparisons among these distinct variants. Following silencing of *PtRTP1* in two wheat near-isogenic lines (NILs; TcLr15 and TcLr36), *TaPR1*, *TaPR2*, *TaPAL*, *TaSOD* and *TaCAT* were all significantly upregulated in wheat plants. In contrast, *TaPR5* displayed completely opposite expression trends between the two NILs: it was significantly downregulated in TcLr15, yet markedly upregulated in TcLr36. These observations suggest that the mechanisms by which PtRTP1 suppresses wheat immune responses may differ across these near-isogenic lines. In future work, we will further screen for interacting partners of PtRTP1 in different NILs and measure physiological parameters including salicylic acid (SA) and ROS in these wheat lines to further elucidate the functional mechanisms of PtRTP1.

In the present study, heterologous expression assays in tobacco cells were performed to characterize the subcellular localization of PtRTP1, revealing that PtRTP1 predominantly localizes to the tobacco cell nucleus. Previous studies have reported that Us-RTP1p is localized to the extrahaustorial matrix and host cytoplasm but is barely detectable in the nucleus of infected plant cells. Moreover, both Uf-RTP1p and Us-RTP1p exist mainly as glycosylated forms in infected leaves [28]. Therefore, subcellular localization results obtained via heterologous expression systems may have inherent limitations, and further investigations are required to dissect the functional mechanisms of PtRTP1. Heterologous expression assays in *N. benthamiana* cells also preliminarily demonstrated that PtRTP1 could suppress BAX-induced PCD. Though these heterologous assays lacked immunoblot validation of protein accumulation and quantitative cell-death measurements, these findings preliminarily suggest that PtRTP1 can repress plant cellular defense responses, though this observation should be interpreted with caution. This result provides preliminary experimental evidence for deciphering the role of PtRTP1 during *P. triticina* infection.

The RTP family constitutes a class of effector proteins. Previous research has demonstrated that Uf-RTP1 exhibits protease-inhibitor activity [30]. Silencing of Pst18363, an RTP1-homologous gene in *P. striiformis*, attenuates the virulence of wheat stripe rust. This effector targets TaNUDX23 to suppress ROS accumulation, yet it cannot directly modulate the Nudix hydrolase activity of TaNUDX23 [31]. This finding is inconsistent with the report that Uf-RTP1 possesses protease-inhibitory activity [30]. To date, the molecular mechanisms underlying other RTP1 homologs remain uncharacterized. In the present study, we only characterized the function of PtRTP1 during *P. triticina* infection. We preliminarily demonstrated that silencing PtRTP1 also attenuated the virulence of *P. triticina* (Figure 6 and Figure 7). Taken together with the role of Pst18363 during *P. striiformis* infection, these results preliminarily suggest that the RTP1 family represents a promising candidate for broad-spectrum disease-management strategies against obligate biotrophic fungi, rather than a validated fungicide target. However, no interacting targets of PtRTP1 were identified in wheat plants in our current study. In future work, we will screen for these host interacting targets of PtRTP1, characterize their biological functions, and elucidate the molecular interaction mechanisms underlying the wheat–*P. triticina* interaction. These efforts will facilitate the comprehensive clarification of the biological functions and systematic mechanisms of the RTP family during the infection of obligate biotrophic fungi.

## 4. Materials and Methods

### 4.1. Materials

Wheat cultivars susceptible to leaf rust, including Thatcher and Zhengzhou 5389, as well as *P. triticina* physiological races PHTP, THKT, THTS, FHHT, KHHT and THSQ were preserved and provided by the Laboratory of Molecular Plant Pathology, Hebei Agricultural University. *A. tumefaciens* competent cell strain GV3101 was purchased from Weidi Biotechnology Co., Ltd. (Shanghai, China, Cat. No. AC1001). The main reagents used in this study were as follows: 2 × Es Taq MasterMix (Cwbiotech, Taizhou, China, Cat. No. CW0690H); high-fidelity DNA polymerase Phanta^®^ Max Super-Fidelity DNA Polymerase (Vazyme Biotech Co., Ltd., Nanjing, China, Cat. No. P505-d1); pMD19-T Vector (TaKaRa, Dalian, China, Cat. No. 6013); EasyScript One-Step gDNA Removal and cDNA Synthesis SuperMix reverse transcription kit (TransGen Biotech Co., Ltd., Beijing, China, Cat. No. AE311-02); PVDF membranes (Coolaber Technology Co., Ltd., Beijing, China, Cat. No. YA1701); UNIQ-10 column Trizol total RNA extraction kit and plant total protein extraction kit (Sangon Biotech Co., Ltd., Shanghai, China, Cat. No. B511321-0100, C510004-0020); mouse anti-GFP primary antibody, goat anti-mouse HRP-conjugated secondary antibody and ECL chemiluminescence detection reagent (TransGen Biotech Co., Ltd., Beijing, China, Cat. No. HT801-01, DW101-01); protein molecular weight marker (Sangon Biotech Co., Ltd., Shanghai, China, Cat. No. C620014-0250).

### 4.2. Cloning and Expression Profiling of PtRTP1 from P. triticina

Thatcher was inoculated with *P. triticina* isolates THTT and FGD. Leaves were sampled at 0, 6, 12, 18, 24, 36, 48, 72 hpi, 5 and 7 dpi to extract total RNA, which was reverse-transcribed into cDNA with the EasyScript one-step kit and stored at °2212; °C. Primers PtRTP1-F/PtRTP1-R were designed from the PtRTP1 ORF (Supplementary Data S1). Target gene fragments were amplified via PCR with Phanta^®^ Max Super-Fidelity DNA Polymerase according to the product instructions. Purified PCR products were ligated into the pMD19-T Vector, followed by transformation into competent cells. Positive transformants were selected and subjected to Sanger sequencing, and clones carrying the correct PtRTP1 sequence were reserved for downstream experiments. qRT-PCR was performed to analyze the transcript levels of PtRTP1 at the aforementioned time points in the two physiological races. The 2^−ΔΔCT^ method was adopted to calculate average relative expression, standard error and statistical significance [31]. Furthermore, statistical analysis of all experimental data was performed using SPSS 22.0 software. One-way analysis of variance (ANOVA) was used for multiple-group comparison, and an independent Student’s *t*-test was applied for pairwise comparison between two groups. All experimental graphs were plotted with GraphPad Prism8.0 software.

### 4.3. Polymorphism Analysis of PtRTP1 Among Different Physiological Races of P. triticina

Urediniospores of *P. triticina* races PHTP, THKT, THTS, FHHT, KHHT, THSQ, THTT and FGD were propagated and harvested on wheat cultivar Zhengzhou 5389. Genomic DNA of these isolates was extracted using the CTAB method [32]. PCR amplification was carried out with primer pair PtRTP1-F/PtRTP1-R (Supplementary Data S1) using DNA templates from distinct races. The amplified fragments were ligated into pMD19-T Vector and transformed into competent Escherichia coli DH5α cells. Six positive clones derived from each race were sent to Sangon Biotech (Shanghai) Co., Ltd. for Sanger sequencing. The sequence data of race BBBD were downloaded from the NCBI database. A total of 13 sequences were obtained. The sequence data were aligned via MEGA X software [33] to analyze the sequence polymorphism of PtRTP1 across tested *P. triticina* races. Homologous protein sequences of PtRTP1 were retrieved from the NCBI database. Multiple sequence alignment of RTP1 homologous proteins was performed using MEGA X software. The phylogenetic tree was constructed via the Maximum Likelihood method with the General Time-Reversible substitution model. Gaps and missing data were partially deleted during tree construction. Branch reliability was evaluated with 1000 bootstrap replicates. Signal peptide prediction was performed using SIGNALP 6.0 (https://services.healthtech.dtu.dk/services/SignalP-6.0/, accessed on 14 July 2026). Conserved cysteine residues and putative N-glycosylation sites within PtRTP1 were characterized, with N-glycosylation sites predicted using the NetNGlyc-1.0 server (https://services.healthtech.dtu.dk/services/NetNGlyc-1.0/, accessed on 14 July 2026).

### 4.4. Validation of the Secretory Function of PtRTP1 Signal Peptide

The yeast signal sequence trap system [34] was utilized to verify secretion activity of the putative PtRTP1 signal peptide. After amplifying the signal peptide fragment with custom primers and ligating it into pSUC2T7M13ORI, the recombinant construct was transformed into invertase-null YTK12 yeast and cultured at 30 °C [35]. Transformants were preliminarily screened on CMD-W medium, and invertase activity was validated via the TTC color reaction, where TTC is reduced into insoluble red TPF [36]. This experiment was repeated twice.

### 4.5. PtRTP1 Suppresses BAX-Triggered Plant PCD

The signal peptide-deleted PtRTP1 fragment was inserted into pGR107 via homologous recombination to generate recombinant pGR107-PtRTP1^Δsp^, which was transformed into Agrobacterium tumefaciens GV3101. Transformed strains were cultured in LB medium supplemented with 25 μg/mL rifampicin and 50 μg/mL kanamycin at 28 °C and 180 rpm for 1–2 days.

The GV3101 strains carrying empty pGR107, pGR107-BAX, pGR107-PtRTP1^△SP^ and pGR107-Avr1b^ΔSP^ were separately washed twice with agroinfiltration buffer, with each suspension adjusted to OD600 = 0.5–0.6. Four-week-old *N. benthamiana* leaves were infiltrated with the bacterial suspensions using needleless 1 mL syringes and incubated under dim light. After 24 h, leaves were infiltrated with GV3101 carrying pGR107-BAX. Cell death lesions on leaves were photographed at 5–7 d post infiltration after complete decolorization with absolute ethanol [37].

### 4.6. Subcellular Localization Assay of PtRTP1

GV3101 strains harboring pGR107-PtRTP1^△SP^ and empty pGR107 were resuspended in agroinfiltration buffer to OD600 = 0.5 and then incubated statically in darkness at 28 °C for 2 h. The bacterial suspensions were infiltrated into leaves of 4-week-old *N. benthamiana*. Infiltrated plants were kept in dark and humid conditions before shifting to dim light 24 h later. Fluorescence observation and imaging were performed under a fluorescence microscope at 48 h post infiltration [38].

### 4.7. Virulence Functional Analysis of PtRTP1

siFi 21 software was used to predict off-target effects based on the cDNA sequence of *PtRTP1*. Based on prediction analyses, the 551–764 bp region (214 bp in total) of the *PtRTP1* sequence was selected as the silencing fragment, and SiFi-software-based predictions revealed no off-target effects for this fragment. The selected silencing fragment was ligated into the pCaBS-γbLIC vector [39]. *A. tumefaciens* EHA105 cultures harboring pCaBS-α, pCaBS-β and either pCaBS-γbLIC, pCaBS-γbPDS or pCaBS-γbLIC-PtRTP1 were mixed at a 1:1:1 ratio and infiltrated into leaves of 4-week-old *N. benthamiana*. *N. benthamiana* leaves were collected 7–10 days post infiltration when obvious chlorosis was observed, and leaf tissues from control and treatment groups were separately used to inoculate wheat seedlings [39].

After successful establishment of BSMV infection in wheat, fresh urediniospores of highly virulent *P. triticina* isolate THTT and weakly virulent isolate FGD were inoculated onto wheat leaves. The response of various leaf rust resistance genes to these *Pt* races was explained by Long and Kolmer (1989) [40] in their proposed nomenclature system that designated the virulence of cultures of *Pt* combinations (infectivity: “0” no uredinium or infection court; “;” no uredinium, but there is necrosis or chlorosis; “1” the uredinium are small and have necrotic spots around them; “2” uredinium are small to medium, with necrosis or chlorosis around them; “3” the uredinium is medium, with or without chlorosis; “4” uredinium is large, without chlorosis and necrosis, often satellite uredinium). Inoculated plants were incubated overnight under dark and humid conditions and then grown at (25 ± 3) °C with a 16 h light/8 h dark photoperiod. Disease phenotypes were recorded and photographed at 12–14 days after rust inoculation. This experiment was carried out with two biological replicates, and a minimum of three leaves were examined for each treatment.

### 4.8. Analysis of Silencing Efficiency of PtRTP1 and Expression Levels of Pathogenesis-Related Genes

Inoculated wheat leaves were sampled at 0, 24, 48 and 120 hpi. Three biological replicates were set for each treatment. Total RNA was extracted and reverse-transcribed into cDNA, which was diluted to 100 ng/µL for subsequent qRT-PCR. The primer pairs QPtRTP1F/QPtRTP1R and reference gene primers PtActinF/PtActinR (Supplementary Data S1) were used to quantify the silencing efficiency of PtRTP1 in control and silenced plants [24]. Another set of primers targeting defense-related genes *TaPR1*, *TaPR2*, *TaPR5*, *TaCAT*, *TaPAL*, *TaSOD* and the internal reference gene *TaEF* (Supplementary Data S1) was used to detect transcript levels of *TaPR1*, *TaPR2*, *TaPR5*, *TaCAT*, *TaSOD* and *TaPAL*. The 2^−ΔΔCT^ method was adopted for data analysis to calculate the average relative expression, standard error and statistical significance of differences [31].

## 5. Conclusions

The ORF of PtRTP1, an effector from *P. triticina*, is 777 bp in length and encodes a 258 aa protein with a 23-residue signal peptide. The precursor protein has a predicted molecular weight of 28.3 kDa and carries two putative N-glycosylation sites. Two polymorphic amino acid sites and nine conserved cysteine residues were identified across different physiological races of *P. triticina*. Transcription of PtRTP1 is upregulated upon wheat infection and peaks at 24 hpi. Results from heterologous expression assays in *N. benthamiana* cells demonstrated that PtRTP1 functions in the nucleus and can suppress BAX-induced PCD. Functional assays verified that PtRTP1 serves as a virulence factor in both tested races THTT and FGD.

## Data Availability

The original contributions presented in this study are included in the article/Supplementary Material. Further inquiries can be directed to the corresponding author.

## Supplementary Materials

The following supporting information can be downloaded at https://www.mdpi.com/article/10.3390/plants15192973/s1, Data S1: Primers used in this study.

## Abbreviations

BXA	Bcl2-associated X
PCD	Programmed cell death
PR	Pathogenesis-related protein genes
SA	Salicylic acid
ROS	Reactive oxygen species
