# Supplementary material for: PtRTP1, an Effector of Puccinia triticina, Contributes to Virulence in Wheat
Source: Plants (Basel). 2026 Sep 29;15(19):2973. doi: 10.3390/plants15192973 (PMC13667110; doi:10.3390/plants15192973)
Supplement: Supplementary file 1 [file plants-15-02973-s001.zip › plants-4514007-supplementary.pdf]

Supplementary Data S1. Primers used in this study

Primer	sequence(5'-3')	application
PtRTP1-F	ATGTCATCGTTTAATTTCTTGTGCA	Cloning of PtRTP1
PtRTP1-R	TCAATTCATGTATCTGTTTCTTTGG	
TaPR1-F	GAGAATGCAGACGCCCAAGC	qRT-PCR
TaPR1-R	CTGGAGCTTGCAGTCGTTGATC	
TaPR2-F	AGGATGTTGCTTCCATGTTTGCCG	
TaPR2-R	AAGTAGATGCGCATGCCGTTGATG	
TaPR5-F	CAAGCAGTGGTATCAACGCAGAG	
TaPR5-R	GTGAAGCCACAGTTGTTCTTGATGTT	
TaPAL-F	TTCGATTTGCCACCAAGTC	
TaPAL-R	GTGCCTTGGAAGTTGCCAC	
TaCAT-F	CCATGAGATCAAGGCCATCT	
TaCAT-R	ATCTTACATGCTCGGCTTGG	
TaSOD-F	CCGAGGTCTGGAACCATCAC	
TaSOD-R	AGCCGAAATCCTTCTCGATCT	
QPtRTP1F	TCCACCTGTGATGTCAACGATTCG	
QPtRTP1R	TTCCGCTATTCTCAACGCCTGAAG	
TaEF-F	TGGTGTCATCAAGCCTGGTATGGT	
TaEF-R	ACTCATGGTGCATCTCAACGGACT	
PtActinF	ATCCACGAGACCACCTACAACT	
PtActinR	CAAGATAGAACCACCAATCCATAC	
HPtRTP1F	AAGTTTAAACCAGGCCGAACGACTTCAA	Silencing assay
HPtRTP1R	ACCACCACCGTCTGTTTCTTTGGAAAGTGAT	
PGRRTP1$^{\Delta sp}$F	CCAGCTAGCATCGATATGGGTTTTCTTCCTCAAGA	Sublocalization
PGRRTP1R	GCTCACCATCCCGGGATTCATGTATCTGTTTCTTT	
Avr1b-F	GTCAGCACCAGCTAGCATCGATACTGAGTACTCCGACGAAAC	Supression of PCD
Avr1b-R	CGCCCTTGCTCACCATCCCGGGGCTCTGATACAGGTGAAAG	
BAX-F	GTCAGCACCAGCTAGCATCGATATGGACGGGTCCGGG	
BAX-R	CGCCCTTGCTCACCATCCCGGGGCCCATCTTCTTCCAG	
SPRTP1F	CCGGAATTCATGGGTTTTCTTCCTCA	Secretion assay
SPRTP1R	CCGCTCGAGTCAATTCATGTATCTGTTTC	
